# Rest–Activity Rhythm Patterns and Their Associations With Depression and Obesity: A Study Using Actigraphy and Human–Smartphone Interactions

**DOI:** 10.1155/da/2617282

**Published:** 2025-03-31

**Authors:** I-Ming Chen, Chen Lin, Guan-Jie She, Hsiang-Chih Chang, Hai-Hua Chuang, Tien-Yu Chen, Yu-Hsuan Lin

**Affiliations:** ^1^Department of Psychiatry, National Taiwan University Hospital, Taipei, Taiwan; ^2^Department of Psychiatry, College of Medicine, National Taiwan University, Taipei, Taiwan; ^3^Department of Biomedical Sciences and Engineering, National Central University, Taoyuan City, Taiwan; ^4^Institute of Population Health Sciences, National Health Research Institutes, Miaoli County, Taiwan; ^5^Department of Community Medicine, Cathay General Hospital, Taipei, Taiwan; ^6^School of Medicine, College of Life Science and Medicine, National Tsing Hua University, Hsinchu, Taiwan; ^7^Department of Psychiatry, Tri-Service General Hospital, School of Medicine, National Defense Medical Center, Taipei, Taiwan

**Keywords:** actigraphy, circadian rhythm, depression, digital phenotyping, human–smartphone interaction, obesity

## Abstract

**Background:** This study aimed to empirically derive subgroups based on both actigraphy- and app-measured rest–activity rhythm (RAR) patterns and investigate the relationship between these profiles and health outcomes, including depression and obesity.

**Methods:** We developed a mobile app, Rhythm, to record human–smartphone interactions and calculate RAR patterns alongside standard actigraphy in 135 participants (mean age: 43.8 ± 12.3 years, 64% women) with and without major depressive disorder and/or obesity. Wrist actigraphy and Rhythm app recorded activity data for at least 4 weeks, totaling 3978 person-days. Person-centered clustering was conducted to identify subgroups based on RAR characteristics, and their associations with clinical outcomes were evaluated using multivariable regression models.

**Results:** Three distinct groups with different RAR patterns were identified based on acrophase, interdaily stability (IS), and intradaily variability (IV), measured by actigraphy and human–smartphone interactions, respectively. The “earlier” group exhibited earlier acrophase both by actigraphy and the app and had lower depressive symptom severity than the other two groups. The “later” group showed a later acrophase and a lower body mass index (BMI) compared to the “earlier” group. The “irregular” group, characterized by higher IV, lower IS, and desynchronized actigraphy- and app-measured acrophase, was associated with higher levels of depressive symptom severity and BMI.

**Conclusions:** Our study highlights the usefulness of human–smartphone interaction patterns in providing a comprehensive understanding of individuals' circadian rhythms beyond standard actigraphy measurements. Identifying distinct RAR profiles based on both actigraphy and app measurements contributes to a better understanding of the associations between circadian disruptions and mental and physical health outcomes.

## 1. Introduction

Human beings are inherently attuned to a diurnal lifestyle, traditionally characterized by wakefulness during daylight and restfulness at night. This natural rhythm, integral to human evolution, plays a critical role in maintaining both mental and physical well-being. However, modern lifestyles have led to significant disruptions in this 24-h cycle, known as rest–activity rhythms (RARs), with profound implications for health. Such disruptions are increasingly linked to a spectrum of conditions, ranging from mood disorders to metabolic diseases [1]. Traditionally, RARs have been assessed through actigraphy, a method that tracks physical activity continuously over time to infer sleep–wake patterns [2]. Yet, this approach captures only a facet of the intricate tapestry of human behaviors that influence circadian rhythms. The advent of digital technologies offers an unprecedented opportunity to enrich our understanding of RARs by incorporating data derived from human interactions with smartphones and other digital devices. These digital footprints, akin to physical activity records, provide supplementary information that extends beyond traditional actigraphy, offering a more nuanced view of daily rhythms. This research endeavors to explore this novel paradigm by integrating traditional actigraphy with patterns of human–smartphone interactions, positing that such an amalgamation could unveil deeper insights into mental health through the lens of RARs. By embracing this comprehensive approach, we aim to capture the complexities of circadian rhythms more effectively, thereby enhancing our understanding of their impact on both mental and physical health.

Since the pioneering work of Witting et al. [3] in 1990, which established a framework for analyzing RARs through key quantifiable parameters such as interdaily stability (IS) and intradaily variability (IV) and applied these insights to study aging and Alzheimer's disease, the scientific community's interest in the role of RARs in neurodegenerative disorders has expanded significantly. This foundational study paved the way for subsequent research exploring the intricate relationship between RAR patterns and various aspects of mental and physical health. For example, Nguyen Ho et al. [4] found that higher fragmentation of 24-h activity rhythms is linked to increased amyloid-beta pathology in APOE4 carriers, Leng et al. [5] associated reduced circadian rhythmicity with an increased risk of Parkinson's disease, and Smagula et al. [6] connected irregular RARs with elevated depression symptoms and cognitive declines in aging populations [4–6]. These studies collectively highlight the profound impact of circadian disturbances across a spectrum of neurodegenerative disorders. Building upon the groundwork laid by Witting et al. [3], the scope of RAR research has broadened to encompass mood disorders such as major depressive disorder [6–12] and bipolar disorders [13–17], marking a significant trend in contemporary studies. In addition to neuropsychiatric disorders, the application of RAR analysis has been extended to examine its connection with obesity, both in community and clinical settings [18–22]. Despite these efforts, the behavioral mechanisms linking obesity and depression through RARs remain elusive, underscoring a gap in our understanding. This gap is particularly notable given the accumulating evidence that RAR characteristics are closely associated with both obesity and depression, indicating a shared pathway that warrants further investigation. Moreover, recent research has employed clustering techniques to identify subgroups within populations based on common RAR profiles, exploring the diverse mental health outcomes across these clusters. This approach has revealed the complex influence of circadian rhythms on health, suggesting that the effects of RARs are multifaceted and cannot be fully captured by a singular measure [6]. These developments underscore the need for continued research to decode the nuanced relationships between RARs, mental illness, and metabolic disease, offering promising avenues for understanding and potentially mitigating the impacts of these conditions.

In recent years, the explosion of digital footprints, especially through human–smartphone interactions, has opened new avenues in the study of circadian rhythms [23, 24]. Beyond traditional actigraphy, which primarily captures physical activity, smartphone data offer insights into social rhythms, cognitive engagement, and the physiological impacts of screen exposure [13–17, 25, 26]. Despite their potential, the full impact of these interactions on circadian rhythm metrics like acrophase, IS, and IV is yet to be fully explored [27]. Incorporating human–smartphone interaction patterns into RAR measures might provide a more clinically meaningful approach to understanding an individual's circadian rhythm in the modern era, as real-time, passively collected data from these interactions can potentially offer an alternative to actigraphy for determining chronotype and RAR indicators [23, 24]. Incorporating human–smartphone interaction patterns into RAR measures can provide a more clinically meaningful approach to understanding an individual's circadian rhythm in the modern era, as real-time, passively collected data from these interactions can potentially offer an alternative to actigraphy for determining chronotype and RAR indicators.

Our study aimed to address gaps in knowledge related to circadian rhythm disruption and its impact on mental and physical health outcomes. Specifically, we aimed to empirically derive subgroups of individuals with similar RAR profiles, which were measured by both standard actigraphy and human–smartphone interaction patterns. We would then characterize the mental and physical health of these subgroups across multiple symptom dimensions. We hypothesized that using both standard actigraphy and human–smartphone interaction patterns to calculate RAR would provide a more comprehensive understanding of circadian rhythm disruption than focusing solely on physical activity measured by standard actigraphy.

## 2. Material and Methods

### 2.1. Study Population

A total of 135 participants (mean age: 43.8 ± 12.3 years, 64% women) were recruited from three psychiatric outpatient clinics, an obesity outpatient clinic, and a workplace health promotion program at seven hospitals in northern Taiwan for the study period between September 2021 and February 2023. Our study sample included individuals with a history of DSM-5-defined major depressive disorder who were followed in psychiatric clinics and were under continuous pharmacological treatment, individuals with obesity who were participating in an obesity outpatient clinic, and health-care professionals who were enrolled in the workplace health promotion program. Specifically, there were 44 obese participants (body mass index [BMI] ≧ 27), 46 participants diagnosed with major depressive disorder by psychiatrists, and 11 participants diagnosed with both major depressive disorder and obesity (BMI≧27). Additionally, 34 participants served as healthy controls, as they had neither major depressive disorder nor obesity. This diverse sample enabled us to examine the associations between RAR profiles and major depressive disorder, obesity, and their comorbid conditions, as well as to capture behaviors that explained variance in mental and physical health distinct from sleep.

In determining our sample size, our study focuses on comparing the clinical outcomes of various RAR patterns, particularly depressive symptoms and obesity. Anticipating three to four RAR pattern groups corresponding to these clinical outcomes, we conducted a sample size analysis using G^*⁣*^*∗*^^Power software [28]. Employing an *F*-test within a fixed effect, omnibus, one-way ANOVA model, we set an alpha error probability of 0.05 and a statistical power of 0.80. With an effect size of 0.3 expected for the three to four groups, the corresponding total sample size ranged from 111 to 128 participants. Additionally, we referenced a study [27] that involved 33 insomnia patients and 33 healthy controls, informing our sample size estimation. Our sample size of 135 participants not only surpasses this reference but also ensures robust statistical power for analyzing complex relationships within our data. Furthermore, the inclusion of 34 healthy controls in this study exceeds previous control group sizes. This larger sample size facilitates a nuanced examination of clinical correlations, incorporating five independent variables (RAR groups, diagnosis, age, gender, and sleep indicators or physical activity level) in line with the “one in ten rule” for regression analysis, thus justifying our sample size selection. Specifically, our sample size exceeds the minimum requirement of 50 participants for regression analysis.

All participants provided written informed consent before taking part in the study. They were asked to install the *Rhythm* app and wear a wrist actigraphy device for a minimum of 4 weeks, resulting in a total of 3978 person-days of data collection. Only participants with Android-operating smartphones were eligible to participate, with the condition that their phones were to be exclusively used by them during the study period. While participants were instructed to wear their actigraphy devices continuously, there was no specific guidance on the placement of their smartphones. The study was approved by the Institutional Review Boards of the National Taiwan University Hospital (IRB No. 202004005RIND), Tri-Service General Hospital (IRB No. B202205050), and the Chang-Gung Memorial Hospital (IRB No. 202002452A3 and 202100434B0A3) and was conducted in accordance with the ethical principles outlined in the Declaration of Helsinki.

### 2.2. Clinical Outcomes

#### 2.2.1. Depressive Symptoms

The Patient Health Questionnaire-9 (PHQ-9) is a widely used, self-administered instrument for detecting depressive symptoms. For each of the nine depressive symptom criteria, subjects indicate the extent to which the symptoms bother them using options like “not at all,” “several days,” “more than half the days,” or “nearly every day.” Each criterion yields a score between 0 and 3, such that the PHQ-9′s total score ranges from 0 to 27. A PHQ-9 score of 10 or greater has a sensitivity of 93% and a specificity of 88% for the diagnosis of major depressive disorder [29]. The diagnostic validity of the Chinese version of PHQ-9 is comparable with clinician-administered [30]. In this study, PHQ-9 data were collected at baseline, week 2, and week 4 of the study period. We found no significant differences between these time points.

#### 2.2.2. Weight Status

All participants had their weight (in kilograms) and height (in centimeters) measured without shoes, following standard protocols. The BMI was calculated by dividing the body weight (in kilograms) by the square of the body height (in meters), resulting in the unit of kg/m^2^. The BMI categories used were overweight, defined as a BMI between 24.0 and 26.9 kg/m^2^, and obesity, defined as a BMI equaled to or greater than 27.0 kg/m^2^ [31]. BMI measurements were taken at the beginning of the study period.

### 2.3. Sleep and Circadian Rhythm Measures: The Use of Mobile App “Rhythm” and Actigraphy

#### 2.3.1. Actigraphy, Physical Activity, and Sleep Indicators

The participants were instructed to wear a research-grade wrist actigraphy device (MiCorTM A100, MiTAC Inc. Taiwan) on their nondominant wrist for a minimum of 4 weeks. The rationale for requiring a minimum of 4 weeks to use the app and wrist actigraphy device was based on our findings that a 28-day measurement period was more effective than the commonly used 7-day period in capturing the link between disrupted circadian rhythms and adiposity [32]. Our app tracked both circadian and social rhythms, and a single 7-day period often failed to fully capture the variability in social rhythms, such as the transition between workdays and weekends, due to individual differences in sleep patterns and behavior. For instance, an individual could have experienced insufficient sleep during the weekdays of the first week and compensated by sleeping longer on the weekend, whereas the same individual might have exhibited normal sleep patterns during the second week. This demonstrated how a single 7-day period could have led to inconsistencies and insufficient data. By tracking the 7-day cycle over 28 days, encompassing three or more cycles, our study provided a more comprehensive and stable assessment of social rhythms. Additionally, human biological rhythms, such as the 28-day menstrual cycle, could have affected weight and body composition, further underscoring the importance of a 28-day measurement period for a holistic evaluation of circadian and biological rhythms and their relationship to adiposity [32, 33].

This device collected data on acceleration across three axes, with an internal sampling rate of 30 Hz. Before being aggregated into epochs, the acceleration data were processed to calculate the Euclidean distance, which represents the magnitude of deviation from a zero point. The data were then bandpass filtered from 0.5 to 3 Hz, and zero values above a predefined threshold were integrated within 2 s. From there, activity counts were derived by averaging the integrated segments over 1 min [2, 34].

The physical activity level of each participant was quantified by using an accelerometer to measure their daily movements. Key features were calculated for each day, including the M10 and L5 values. The M10 and L5 represented the 10 h of the day when the participant was most active and the 5 h when they were least active, respectively. These are widely recognized indicators of a person's circadian activity patterns [35, 36]. To determine the M10, a 10-h moving average was used to estimate the period of the day with the highest average acceleration, which was considered to be the participant's overall physical activity level.

To determine the sleep and wake times, the standard Cole–Kripke algorithm [34] was applied to the activity count data with slight modifications. The algorithm categorized data into rest and active states based on a weighted sum of the current minute and contiguous minutes to minimize the impact of sudden changes in activity levels that could compromise the categorization. Sleep onset was defined as a period of 20 consecutive epochs with zero acti-count, while wake time was defined as a period of 8 consecutive epochs with non-zero acti-count. The daily sleep indicators included sleep onset, wake time, wake after sleep onset (WASO), and total sleep time (TST). The algorithm was run on MATLAB software (MathWorks, Natick, USA) and used pre-existing codes.

#### 2.3.2. Human–Smartphone Interaction Patterns

The app, *Rhythm*, was designed to collect data on smartphone usage by tracking three key variables: screen on/off events, notifications, and the app being used [24, 37]. The data were collected in the background without interfering with the smartphone operation or affecting battery life (less than 1%). We developed an innovative approach to analyze human–smartphone interaction patterns as a proxy for activity levels, mirroring the methodology typically employed in wrist actigraphy. This involved defining each instance of smartphone use from the moment the screen was turned on until it was subsequently turned off as a single episode. We then measured the intensity of smartphone interaction by counting the number of app uses per minute. To address the sporadic nature of smartphone use and reduce the prevalence of periods with zero counts, we aggregated these counts into 5-min epochs. This adjustment provided a more stable and meaningful measure of smartphone activity levels in our previous studies [27, 32]. To interpret these data within the context of circadian rhythms, we employed a single-component single cosinor model. This model is particularly suited for analyzing phenomena that exhibit regular, predictable cycles, such as the 24-h circadian rhythm inherent in human physiology. By fitting the model to the time series of app counts, we were able to delineate the active and inactive phases of participants' daily routines. The active phase was characterized by higher app counts, peaking at the acrophase, while the inactive phase, indicative of potential sleep periods, was identified by a significant drop in app usage, reaching its lowest point at the nadir. By quantifying human–smartphone interactions in this manner and mapping them onto a near-24-h cycle, we effectively mimicked the actigraphy-derived activity data, allowing us to estimate the circadian rhythm of smartphone users.

#### 2.3.3. RAR Measures

**Figure 1:**
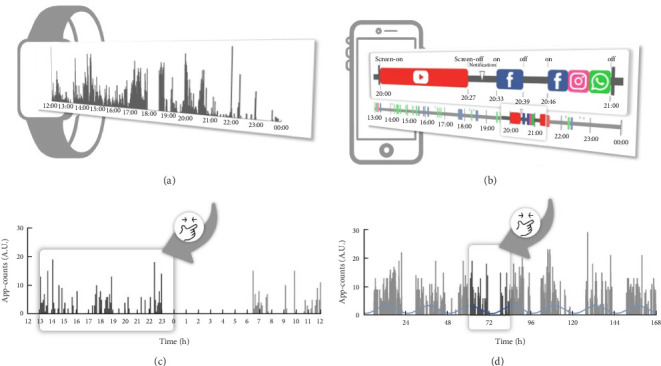
Methods for calculating rest–activity rhythms of physical activity and human–smartphone interactions. (A) Physical activity data measured by wrist actigraphy. (B) Human–smartphone interaction patterns obtained by tracking timestamps of three key variables: screen on/off events, notifications, and the app being used recorded by the *Rhythm* app. (C) The algorithm converts these timestamps into “app-counts,” analogous to “acti-counts” in physical activities. The app-counts exhibit diurnal fluctuation in the 24-h data. (D) The 7-day (168 h) data from either the *Rhythm* app or actigraphy enables the calculation of rest–activity rhythms.

We used parametric cosinor analysis and nonparametric method to calculate circadian rhythm indicators based on the 4-week dataset of app-counts or acti-counts (Figure 1). To quantify the rhythm of activity or app usage data, we further used the hourly averaged acti-counts and app-counts and fitted by a “cosinor analysis” with a 24-h sinusoidal function, $\alpha \left(t\right)=M+Acos\left(2\pi \times \frac{t-\varphi }{24}\right)+\varepsilon$, in which “*M*” was the midline estimated statistic of rhythm (MESOR), “*φ*” was the acrophase (the time of the peak activity of the 24-h rhythm) and *ε* was the unexplained residual. The “*A*” represented coefficients of the 24-h of the cosinor fitted model and was used to estimate the amplitude of the daily rhythm (the difference between peak amplitude and MESOR).

A nonparametric method was used to calculate the circadian rhythm indicators IS and IV [3]. IS quantified the rhythm's stability between days, that is, the coupling strength of the rhythm to the supposedly stable environmental factors. It could vary between 0 and 1, with higher values indicating more stable daily rhythms.

  $$\begin{array}{c} IS=\frac{N\sum_{h=1}^{p}{\left(\overset{―}{x_{h}}-\overset{―}{x}\right)}^{2}}{p\sum_{i=1}^{N}{\left(x_{i}-\overset{―}{x}\right)}^{2}}, \end{array}$$

where IV indicated the rhythm's fragmentation, that is, the frequency and extent of transitions between rest and activity. It could vary roughly between 0 and 2, with higher values indicating higher fragmentations.

  $$\begin{array}{c} IV=\frac{N\sum_{i=2}^{N}{\left(x_{i}-x_{i-1}\right)}^{2}}{\left(N-1\right)\sum_{i=1}^{N}{\left(x_{i}-\bar{x}\right)}^{2}}. \end{array}$$

This study used both the aforementioned nonparametric indicators and one cosinor analysis indicator, the acrophase. In cosinor-based rhythmometry, which includes the MESOR (a rhythm-adjusted mean), amplitude (a measure of half the extent of predictable variation within a cycle), and acrophase (a measure of the time of overall high values recurring in each cycle), only the acrophase has the unit of time and is comparable with the app-defined and actigraphy indicators. Meanwhile, the unit of the MESOR and amplitude are app-counts or acti-counts, so MESOR_app_ and MESOR_act_ are not comparable. In addition, we did not use the relative amplitude in the nonparametric method because the relative amplitude is the ratio of the differences between the most active 10 continuous hours (M10) and the least active 5 continuous hours (L5) over the summation of M10 and L5. However, the L5 measured by human–smartphone interaction patterns was almost always 0, unlike physical activity where there is still a weak signal during L5. Therefore, the relative amplitude measured by the app was almost always very close to 1 in all individuals and could not be used as a parameter to distinguish between groups.

### 2.4. Statistical Analysis

We conducted person-centered clustering using finite normal mixture modeling, fit with the expectation-maximization algorithm, implemented using the R Software package “MClust” [38]. The RAR variables, measured via both actigraphy and mobile app, described above were entered, and we considered models that had different numbers of groups and covariance structures. We used the Bayesian Information Criterion to select the optimal model but specified a priori that we would reject solutions that included groups composed of <10% of the sample. Using the optimal model identified with Bayesian Information Criterion, for descriptive purposes, we evaluated how the derived groups differed in terms of their average RAR measures.

We analyzed continuous variables, such as age and BMI, using one-way ANOVA and Scheffe post hoc analysis, while categorical variables, like gender, were assessed with the Chi-square test. Our investigation then focused on discerning differences in PHQ-9 scores and BMI among the RAR groups, employing one-way ANOVA. We only proceeded with multivariable analysis for clinical outcomes that demonstrated significant differences (*p*  < 0.05) across the RAR groups. This approach aimed to control for potential confounders, including variations in sleep and activity levels, as well as demographic factors like age and gender, ensuring a more accurate interpretation of the data.

We utilized multivariable regression models to examine the impact of physical activity level (M10) and specific night-time sleep parameters (WASO and TST) on the relationship between the RAR group and clinical outcomes, such as BMI and PHQ-9 scores. Age, gender, and diagnosis were consistently adjusted for in all models. Additionally, our base models were adjusted to incorporate diagnostic information, thus considering additional dimensions beyond the outcome variable. For instance, when analyzing depressive symptom scores as the outcome/dependent variable, we included BMI as an independent variable; conversely, when BMI was the outcome/dependent variable, depressive symptom scores (PHQ-9) were incorporated as an independent variable. To facilitate comparative analyses between groups, these regression models were applied, utilizing the irregular group as the reference, to systematically compare the later and earlier groups and elucidate their differential effects on the aforementioned clinical outcomes.

## 3. Results

**Figure 2:**
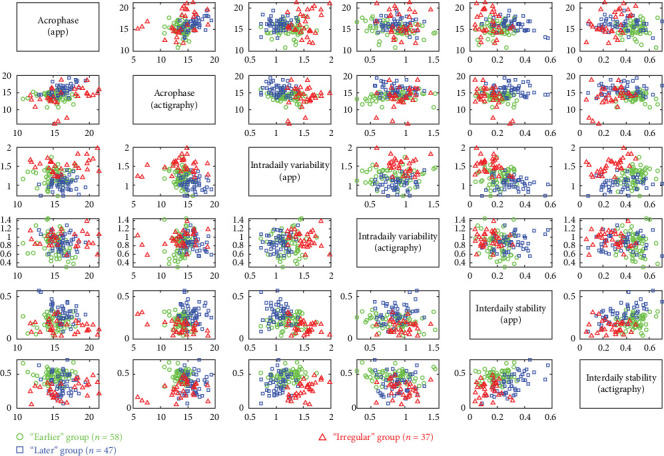
Scatter plots illustrate variations in RAR variables among clusters. Each scatter plot depicts pairs of RAR variables stratified by the derived RAR groups. In each plot, individual data points represent participants, with their respective group affiliations indicated in the legend. RAR, rest–activity rhythm.

The Bayesian Information Criterion indicated that the optimal solution consisted of three groups with ellipsoidal, equal volume, and orientation. These groups, derived from the solution, exhibited significant differences in average RAR characteristics, as depicted in Figure 2 and Table 1. The clustering methodology aimed to maximize mean differences across empirically derived groups. Table 1 illustrates how participants were grouped based on their RAR profiles, assessed through both actigraphy and human–smartphone interactions using six parameters. The optimal solution from the clustering approach resulted in three distinct groups characterized by their morningness-eveningness profiles, defined by acrophase, and the extent of circadian rhythm disruption, defined by IS and IV. These three groups were labeled as “earlier” (*n* = 58), “later” (*n* = 47), and “irregular” (*n* = 30), respectively.

At that point, we reexamined whether the results truly represented distinct RAR phenotypes across diagnoses or if they effectively differentiated between patients with obesity, major depressive disorder, and healthy controls. Among the 44 participants with obesity, the earlier, later, and irregular RAR pattern groups comprised 26, 6, and 12 individuals, respectively. In the group of 46 participants with major depressive disorder, the earlier, later, and irregular RAR pattern groups included 10, 27, and 9 individuals, respectively. Among the 11 participants with both obesity and major depressive disorder, the earlier, later, and irregular RAR pattern groups consisted of 3, 6, and 12 individuals, respectively. Within the 34 healthy controls, the earlier, later, and irregular RAR pattern groups encompassed 19, 8, and 7 individuals, respectively. Importantly, none of these RAR phenotypes within the diagnostic groups constituted less than 10% of the sample, aligning with the principle stated earlier in our statistical approach, which utilized the Bayesian Information Criterion to select the optimal model while precluding solutions comprising groups comprising <10% of the sample.

The participants in the three groups were demographically similar, but they had significant differences in their RAR characteristics. The “earlier” group exhibited an earlier acrophase as measured by both actigraphy and the app, while the “later” group showed a later acrophase based on the same measures. In the receiver operating characteristic analysis, the cutoff points for acrophase measured by actigraphy and the app were 14:44:06 (PM) and 15:59:42 (PM), respectively, distinguishing the “earlier” group from the “later” group. Similarly, the cutoff points for sleep time and wake time were 23:49:30 (PM) and 7:26:57 (AM), respectively, between the two groups. In contrast, the “irregular” group demonstrated unstable RAR as measured by both the app and actigraphy. This group had the lowest IS, with IS measured by the app (IS_app_) below 0.23 and by actigraphy (IS_act_) below 0.38, as well as the highest IV, with IV measured by the app (IV_app_) exceeding 1.41.

**Figure 3:**
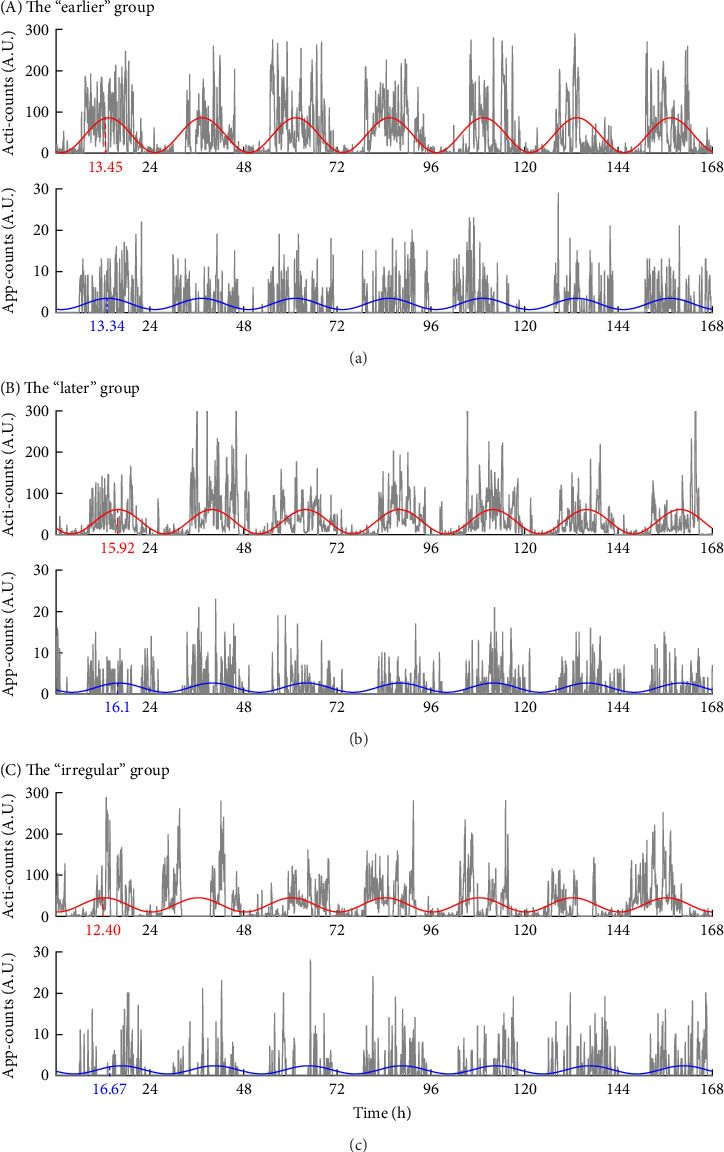
Rest–activity rhythm patterns measured by actigraphy and *Rhythm* app for 7 days (168 h) in each group. (A) Individuals from the “earlier” group with earlier acrophase measures in both actigraphy (13.45, i.e., 13:27 PM) and human–smartphone interaction patterns (13.34). (B) Individuals from the “later” group with a later acrophase from both measures (15.92 and 16.10, respectively). (C) Individuals from the “irregular” group showing irregular rest–activity rhythm patterns, indicated by lower interdaily stability (IS) measured by actigraphy and Rhythm app, high intradaily variability (IV) measured by the app, and the greatest inconsistency between actigraphy- and app-measured acrophase (12.40 and 16.67, respectively).

Besides, the “irregular” group showed an asynchronous rhythm between physical activity and human–smartphone interaction patterns, with the largest differences in acrophase between actigraphy and app (2.82 ± 3.24 h). Participants with RAR characteristics similar to their group's mean values are illustrated in Figure 3. Age, sex, and WASO did not significantly differ among the three groups. However, the “later” group had significantly longer TST than the “earlier” group by 49.1 ± 14.8 min.

In addition, Table 1 shows the analysis to include the timing of the most active 10-h period (M10) and the least active 5-h period (L5), measured by both actigraphy and human–smartphone interactions. The comparative data reveal that the “later” group exhibits a delayed timing in both M10 and L5 intervals compared to the earlier group, mirroring the delayed acrophase observed in the same cohort.

For PHQ-9 scores, although the one-way ANOVA indicated significant differences among the groups (*p* = 0.022), the post hoc analysis did not show significant differences when comparing the groups individually; hence, no specific comparisons were noted. A similar situation was observed in the BMI comparisons; despite a significant difference in the one-way ANOVA test (*p* = 0.049), the post hoc analysis did not reveal significant differences among the individual groups, and thus no comparisons were noted. Given that factors other than RARs, such as TST, WASO, and other potential variables, could influence the differences observed in the clinical outcomes among the groups, we controlled for these potential confounders in subsequent analyses, as detailed in Table 2 to provide a more nuanced understanding of the group differences.

Table 2 presents the diagnosis-, age- and sex-adjusted models to compare the clinical outcomes, PHQ-9 scores, and BMI among the three groups. The “irregular” group, which had the most unstable RAR, had significantly higher PHQ-9 scores than the “earlier” group, while their BMI was higher than the “later” group. We also controlled for M10, TST, and WASO to examine the independence of the RAR group-symptom dimension associations from physical activity level, sleep continuity, and duration. These findings indicate that the observed differences in PHQ-9 scores and BMI among the groups persist, even after considering these factors.

## 4. Discussion

The findings of this study were grossly consistent with previous studies that used actigraphy to classify individuals based on their RAR profiles measured by actigraphy [6, 39] and extended these findings by incorporating human–smartphone interaction data to assess RAR in mental or social activities. In this study, we primarily interpreted circadian rhythms using standard actigraphy measures but used the RAR measured by the app as supplementary evidence. We grouped participants based on their RAR profiles, as measured by actigraphy and the app, and named them according to their consistency across both indicators, resulting in three groups: “earlier,” “later,” and “irregular.” In analyzing the differences among the three groups, as illustrated in Table 1 and visualized in Figure 2, we identified shared RAR patterns that were not discernible through a single metric alone. For instance, in the IV_act_, there were no statistically significant differences between the “later” group, the “earlier” group, and the “irregular” group (Table 1). Consistent with previous studies that linked RAR profiles with psychiatric symptoms [6, 9, 12, 39, 40], our study found that the later chronotype and irregular circadian rhythm groups had higher depressive symptom (PHQ-9) scores than the earlier group. Even though the earlier group had the most regular circadian rhythm, as indicated by the highest IS_act_, their human–smartphone interaction RAR was less regular than the “later” group, with lower IS_app_ and higher IV_app_. The TST of the “earlier” group was also significantly shorter than that of the “later” group (7.10 h versus 7.91 h, *p*  < 0.001). As a result, the earlier and irregular groups had a significantly higher BMI and a higher proportion of obesity (BMI ≧ 27) than the “later” group, which had the longest TST and most stable RAR_app_ with higher IS_app_ and lower IV_app_. This combined analysis of actigraphy and human–smartphone interaction patterns highlights the intricate RARs associated with mental and physical health indicators, offering a more comprehensive understanding than what is provided by traditional actigraphy measures alone.

Our analysis, as detailed in Figures 2 and 3, demonstrates that incorporating smartphone data allows us to identify unique behavioral patterns that actigraphy alone cannot capture. The “irregular” group emerged with distinct circadian rhythm disruptions, characterized by lower IS, higher IV, notable acrophase discrepancies between actigraphy and app measurements (2.82 ± 3.24 h), and also presented with the highest levels of depressive symptoms and obesity. Aligning with prior research [6–12, 39], our findings affirm the link between disrupted RAR and major depressive disorder and introduce a novel insight into the asynchrony between app and actigraphy measurements as potential indicators of broader health implications, particularly obesity. This suggests that such discrepancies might indicate a mental–physical “jetlag,” potentially affecting overall health [41]. Further exploration is needed to understand whether these asynchronies are driven by smartphone usage behaviors or reflect inherent characteristics detectable through smartphones, as well as the implications of such differences for mental activity proxies.

Our study utilized the RAR of the human–smartphone interaction method to address the limitations of previous studies that solely relied on actigraphy to measure RAR. In our subgroup analysis, the irregular group had lower overall IS and higher IV compared to the other two groups. Although the IV_act_ was the highest in the irregular group, the difference was not statistically significant (*p* = 0.073) among the three groups. However, the IV_app_ was the highest in the irregular group with statistical significance (*p*  < 0.001). In comparison to the previous RAR measured by only actigraphy, the earlier group was labeled as the earlier/robust group due to the earlier acrophase measured by actigraphy, which often co-occurred with a more stable circadian rhythm, as evidenced by lower IV, higher IS, and higher relative amplitude [6, 12, 39]. However, in our subgroup analysis that used both actigraphy and human–smartphone interaction patterns, the irregular group did not have a significantly different acrophase measured by actigraphy compared to the earlier group but had a significantly later acrophase measured by the *Rhythm* app and the least stable circadian rhythm, with the lowest IS_app_ and highest IV_app_, among the three groups. The “earlier” group had the most stable circadian rhythm measured by actigraphy, with the highest IS_act_, but the “later” group had the most stable circadian rhythm measured by human–smartphone interaction patterns, with significantly higher IS_app_ and lower IV_app_ compared to the “earlier” group.

This study can address the unmet need in current clinical practice and related research. The most common sleep assessments in general clinical practice are sleep–wake time and sleep duration. In this study, only the finding that the “earlier” group sleeps and wakes earlier with shorter sleep time could be drawn. It was difficult to determine the impact on depressive symptoms or obesity, especially for the “irregular” group with higher levels of depression and obesity that could not be identified by such assessment. If the actigraphy measurement was applied, the characteristics of the “irregular” group could be identified based on the lower IS_act_, but there was no significant difference in IV_act_ compared to the other two groups. Therefore, adding the IS_app_ and IV_app_, which could show significant differences between the groups, could enhance the judgment of actigraphy-measured RAR. In addition, the actigraphy-measured acrophase of the “irregular” group did not differ significantly from that of the “earlier group,” and it was necessary to measure the acrophase of human–smartphone interaction patterns to distinguish the “irregular” group from this study, where the “irregular” group showed greater asynchronous rhythm between physical activity and human–smartphone interaction patterns.

Our study focused on the relationship between circadian rhythm disruptions, depression, and obesity due to the strong evidence linking these conditions. Circadian abnormalities, regulated by key clock genes such as CLOCK and BMAL1, are well-documented in depressive disorders and significantly impact metabolic processes associated with obesity [42]. Moreover, disturbances in sleep–wake cycles, a core component of circadian rhythms, are central to the pathology of depression [43]. While anxiety is frequently comorbid with depression, its connection to circadian rhythms is more variable and less mechanistically defined, making it less suitable for the focused analysis conducted in this study.

It is important to consider the limitations of our study when interpreting the results. First, due to the cross-sectional design of our study, we could not establish a causal relationship between RAR and health outcomes. While we identified subgroups with different RAR profiles, it was unclear whether these profiles were a cause or consequence of mental and physical health outcomes. Longitudinal studies are needed to further investigate these associations and determine potential mechanisms. Second, although our sample included individuals from a workplace health promotion program, we did not have a reference group with low BMI and low depressive symptoms, which limited our ability to compare results. Third, while we derived RAR profiles from human–smartphone interaction patterns, the meaning and validity of these profiles, such as “mental activities” or “social activities,” require further exploration. Specifically, we observed differences in rhythms between mental and physical activity, but it was unclear whether these differences were due to smartphone behaviors or were intrinsic properties captured by the device. Fourth, the Rhythm app we used only works with the Android operating system. Future studies should develop versions for other operating systems, such as iOS and Windows, to increase the generalizability of our findings. Furthermore, although the PHQ-9 self-reported scale was used to assess depressive symptoms, which is subject to recall bias, all diagnoses of major depressive disorder were independently confirmed by psychiatrists according to DSM-5 criteria. This step ensures that our findings on depressive symptoms are underpinned by robust clinical assessments, thereby mitigating the limitations inherent to self-reported data. Fifth, while our sample size aligns with the requirements outlined in our sample size estimation, the recruitment strategy aimed to encompass a certain proportion of individuals with obesity, major depressive disorder, both conditions and healthy controls. Consequently, participants were recruited from various settings, including obesity clinics, psychiatric outpatient clinics, and other healthcare facilities. This convenience sampling method poses methodological limitations, as the sample may not be fully representative of the broader population. To enhance the generalizability of our findings, future research should endeavor to recruit more representative samples from larger, longitudinal cohorts, thereby bolstering the robustness and applicability of our study's conclusions. Although pharmacological treatments, including antidepressants and benzodiazepines, may influence circadian rhythms, existing evidence suggests that these effects are generally subtle or inconsistent. For instance, serotonin reuptake inhibitors and tricyclic antidepressants tend to stabilize circadian rhythms by modulating rapid eye movement sleep or advancing suprachiasmatic nucleus phases, while BZDs have minimal impact on circadian rhythm parameters [44, 45]. If medication effects were significant, they would likely reduce variability rather than produce the pronounced disruptions observed in the “irregular” group. Furthermore, our use of a multimodal approach combining actigraphy and human–smartphone interactions provides robust data that minimizes potential biases introduced by medications. Nevertheless, future studies should include detailed medication data to better account for their potential influence on RARs. Finally, we acknowledge that such data (e.g., polysomnography) could provide additional insights into sleep patterns and quality; however, our study was designed to focus on noninvasive measures using actigraphy and smartphone interactions.

## 5. Conclusions

In conclusion, our study highlights the usefulness of human–smartphone interaction patterns in providing a comprehensive understanding of individuals' circadian rhythms beyond standard actigraphy measurements. Our results indicate that individuals with irregular circadian rhythms exhibit higher IV and lower IS, as measured by both actigraphy and human–smartphone interaction patterns. Additionally, the irregular circadian rhythm group demonstrated the most desynchronized acrophase between physical activity and human–smartphone interaction patterns. The irregular circadian rhythm group was also associated with more depressive symptoms and higher levels of obesity. Given the RARs are modifiable, our findings provide information on the particular RAR characteristics that may be monitored or targeted in future interventions. Future studies with larger and more diverse populations are necessary to confirm our findings and explore the clinical implications of using human–smartphone interaction patterns in circadian rhythm assessment.

## Figures and Tables

**Table 1:** Demographics and characteristics by groups divided by rest–activity rhythm patterns.

	“Earlier” group (*n* = 58)	“Later” group (*n* = 47)	“Irregular” group (*n* = 30)	*χ* ^2^ or *F*	*p*-Value	Comparison
Gender	—	—	—	0.254	0.881	—
Female (percentage)	36 (62.1)	31 (66.0)	20 (66.7)	—	—	—
Age	42.9 (9.2)	42.5 (12.8)	47.5 (15.9)	1.766	0.175	—
Rest–activity rhythms						
Acrophase^a^ (actigraphy)	13.85 (1.13)	15.85 (1.49)	13.71 (2.99)	19.704	<0.001	L > I, E
Acrophase^a^ (app)	15.16 (1.44)	16.23 (1.30)	16.53 (2.74)	7.672	0.001	L, I > E
Interdaily stability (actigraphy)	0.47 (0.08)	0.36 (0.14)	0.27 (0.11)	33.147	<0.001	E > L > I
Interdaily stability (app)	0.21 (0.07)	0.31 (0.10)	0.14 (0.07)	37.645	<0.001	L > E > I
Intradaily variability (actigraphy)	0.84 (0.26)	0.88 (0.18)	0.95 (0.18)	2.670	0.073	—
Intradaily variability (app)	1.23 (0.20)	1.05 (0.16)	1.52 (0.19)	59.899	<0.001	I > E > L
M10 timing (actigraphy)^a^	13.27 (1.18)	14.97 (1.48)	13.82 (1.65)	19.430	<0.001	L > I, E
M10 timing (app)^a^	14.02 (2.01)	15.25 (1.44)	13.28 (2.12)	11.447	<0.001	L > I, E
L5 timing (actigraphy) ^a^	2.79 (0.90)	4.36 (3.23)	3.65 (1.84)	6.869	0.001	L > E
L5 timing (app)^a^	5.37 (7.38)	3.38 (1.31)	8.34 (7.98)	5.909	0.003	I > L
Sleep indicators						
Sleep onset^a^	23.38 (0.82)	24.21 (1.23)	23.51 (1.37)	7.903	0.001	L > E, I
Wake time^a^	6.60 (0.85)	8.33 (1.46)	7.19 (1.54)	24.703	<0.001	L > E, I
Wake after sleep onset (min)	7.5 (9.7)	12.1 (22.8)	15.6 (26.3)	1.864	0.159	—
Total sleep time (min)	425.8 (55.8)	474.8 (84.4)	445.5 (91.9)	5.504	0.005	L >E
Clinical outcomes						
PHQ9 score	4.0 (5.8)	6.8 (5.4)	6.9 (6.1)	3.945	0.022	—
Body mass index (kg/m^2^)	26.7 (6.0)	24.4 (4.5)	26.9 (5.4)	3.080	0.049	—
BMI ≧ 27 (percentage)	29 (50.0)	12 (25.5)	14 (46.7)	6.999	0.030	E > L

*Note:* Continuous variables, such as age and BMI, were analyzed using one-way ANOVA, with standard deviations presented in parentheses alongside mean values. For categorical variables, such as gender, the Chi-square test was employed. *p*-Values were derived from the statistical tests conducted, namely the one-way ANOVA and the Chi-square test. For instances where *p*  < 0.05, further post hoc analysis was performed. The results of these post hoc analyses are detailed in the Comparison section. For example, in the one-way ANOVA, significant differences were observed among the three groups regarding sleep onset. The post hoc analysis revealed that the “later” group had a significantly later sleep onset compared to both the “earlier” and the “irregular” groups. However, no significant difference was found between the “earlier” and “irregular” groups in the post hoc analysis, leading to a notation in the comparison as “L >E, I.”

Abbreviations: E, the “earlier” group; I, the “irregular” group; L, the “later” group.

^a^Times are in day decimal time, for example, 23.50 = 23:30 (PM).

**Table 2:** Association of RAR group with depressive symptoms and BMI after separate for sleep measures and each other.

	“Irregular” group			“Later” group			“Earlier” group		
				*β* (SE)	Test-statistic	*p*-Value	*β* (SE)	Test-statistic	*p*-Value
Models of BMI									

Base model	Reference			−0.266 (0.114)	2.334	0.022	−0.012 (0.109)	0.111	0.912
Base model + WASO				−0.276 (0.111)	2.495	0.015	−0.034 (0.106)	0.320	0.750
Base model + TST				−0.311 (0.110)	2.837	0.006	0.022 (0.101)	−0.218	0.828
Base model + M10				−0.254 (0.113)	2.244	0.028	0.036 (0.109)	−0.330	0.742

Models of PHQ9									

Base model	Reference			−0.030 (0.121)	0.249	0.804	−0.244 (0.110)	2.229	0.029
Base model + WASO				−0.028 (0.120)	0.234	0.816	−0.218 (0.110)	1.974	0.052
Base model + TST				−0.041 (0.122)	0.337	0.737	−0.253 (0.111)	2.273	0.026
Base model + M10				−0.016 (0.124)	0.129	0.897	−0.304 (0.115)	2.638	0.010

*Note*: All models are adjusted for age and sex. M10: the maximum 10 h of physical activity within a 24-h period.

Abbreviations: BMI, body mass index; RAR, rest–activity rhythm; TST, total sleep time; WASO, wake after sleep onset.

## Data Availability

The authors cannot make this study's raw data publicly available due to restrictions imposed by informed consent from participants in this study, which has been approved by the Institutional Review Boards of the National Taiwan University Hospital, Tri-Service General Hospital, and the Chang-Gung Memorial Hospital. Data are available from the Institutional Review Board of the National Taiwan University Hospital, Tri-Service General Hospital, and the Chang-Gung Memorial Hospital for researchers who meet the criteria for access to confidential data.

## References

[1] Allada R., Bass J. (2021). Circadian Mechanisms in Medicine. *New England Journal of Medicine*.

[2] Ancoli-Israel S., Cole R., Alessi C., Chambers M., Moorcroft W., Pollak C. P. (2003). The Role of Actigraphy in the Study of Sleep and Circadian Rhythms. *Sleep*.

[3] Witting W., Kwa I. H., Eikelenboom P., Mirmiran M., Swaab D. F. (1990). Alterations in the Circadian Rest-Activity Rhythm in Aging and Alzheimer’s Disease. *Biological Psychiatry*.

[4] Nguyen Ho P. T., Hoepel S. J. W., Rodriguez-Ayllon M., Luik A. I., Vernooij M. W., Neitzel J. (2024). Sleep, 24-Hour Activity Rhythms, and Subsequent Amyloid-*β* Pathology. *JAMA Neurology*.

[5] Leng Y., Blackwell T., Cawthon P. M., Ancoli-Israel S., Stone K. L., Yaffe K. (2020). Association of Circadian Abnormalities in Older Adults With an Increased Risk of Developing Parkinson Disease. *JAMA Neurology*.

[6] Smagula S. F., Zhang G., Gujral S. (2022). Association of 24-Hour Activity Pattern Phenotypes With Depression Symptoms and Cognitive Performance in Aging. *JAMA Psychiatry*.

[7] Luik A. I., Zuurbier L. A., Hofman A., Van Someren E. J. W., Tiemeier H. (2013). Stability and Fragmentation of the Activity Rhythm Across the Sleep-Wake Cycle: The Importance of Age, Lifestyle, and Mental Health. *Chronobiology International*.

[8] Maglione J. E., Ancoli-Israel S., Peters K. W. (2014). Depressive Symptoms and Circadian Activity Rhythm Disturbances in Community-Dwelling Older Women. *The American Journal of Geriatric Psychiatry*.

[9] Robillard R., Hermens D. F., Naismith S. L. (2015). Ambulatory Sleep-Wake Patterns and Variability in Young People With Emerging Mental Disorders. *Journal of Psychiatry and Neuroscience*.

[10] Smagula S. F. (2016). Opportunities for Clinical Applications of Rest-Activity Rhythms in Detecting and Preventing Mood Disorders. *Current Opinion in Psychiatry*.

[11] Smagula S. F., Ancoli-Israel S., Blackwell T. (2015). Circadian Rest–Activity Rhythms Predict Future Increases in Depressive Symptoms Among Community-Dwelling Older Men. *The American Journal of Geriatric Psychiatry*.

[12] Smagula S. F., Boudreau R. M., Stone K. (2015). Latent Activity Rhythm Disturbance Sub-Groups and Longitudinal Change in Depression Symptoms Among Older Men. *Chronobiology International*.

[13] Alloy L. B., Boland E. M., Ng T. H., Whitehouse W. G., Abramson L. Y. (2015). Low Social Rhythm Regularity Predicts First Onset of Bipolar Spectrum Disorders Among at-Risk Individuals With Reward Hypersensitivity. *Journal of Abnormal Psychology*.

[14] Frank E., Kupfer D. J., Thase M. E. (2005). Two-Year Outcomes for Interpersonal and Social Rhythm Therapy in Individuals With Bipolar I Disorder. *Archives of General Psychiatry*.

[15] Malkoff-Schwartz S., Frank E., Anderson B. (1998). Stressful Life Events and Social Rhythm Disruption in the Onset of Manic and Depressive Bipolar Episodes: A Preliminary Investigation. *Archives of General Psychiatry*.

[16] Shen G. H. C., Alloy L. B., Abramson L. Y., Sylvia L. G. (2008). Social Rhythm Regularity and the Onset of Affective Episodes in Bipolar Spectrum Individuals. *Bipolar Disorders*.

[17] Sylvia L. G., Alloy L. B., Hafner J. A., Gauger M. C., Verdon K., Abramson L. Y. (2009). Life Events and Social Rhythms in Bipolar Spectrum Disorders: A Prospective Study. *Behavior Therapy*.

[18] Cespedes Feliciano E. M., Quante M., Weng J. (2017). Actigraphy-Derived Daily Rest–Activity Patterns and Body Mass Index in Community-Dwelling Adults. *Sleep*.

[19] Fárková E., Schneider J., Šmotek M. (2019). Weight Loss in Conservative Treatment of Obesity in Women is Associated With Physical Activity and Circadian Phenotype: A Longitudinal Observational Study. *BioPsychoSocial Medicine*.

[20] Laermans J., Depoortere I. (2016). Chronobesity: Role of the Circadian System in the Obesity Epidemic. *Obesity Reviews*.

[21] Li J., Vungarala S., Somers V. K., Di J., Lopez-Jimenez F., Covassin N. (2022). Rest-Activity Rhythm is Associated With Obesity Phenotypes: A Cross-Sectional Analysis. *Frontiers in Endocrinology*.

[22] Qian J., Martinez-Lozano N., Tvarijonaviciute A., Rios R., Scheer F. A. J. L., Garaulet M. (2021). Blunted Rest-Activity Rhythms Link to Higher Body Mass Index and Inflammatory Markers in Children. *Sleep*.

[23] Borger J. N., Huber R., Ghosh A. (2019). Capturing Sleep-Wake Cycles by Using Day-to-Day Smartphone Touchscreen Interactions. *npj Digital Medicine*.

[24] Lin Y.-H., Wong B.-Y., Pan Y.-C., Chiu Y.-C., Lee Y.-H. (2019). Validation of the Mobile App-Recorded Circadian Rhythm by a Digital Footprint. *JMIR mHealth and uHealth*.

[25] Brown L. F., Reynolds C. F., Monk T. H. (1996). Social Rhythm Stability following Late-Life Spousal Bereavement: Associations With Depression and Sleep Impairment. *Psychiatry Research*.

[26] Haynes P. L., Ancoli-Israel S., McQuaid J. (2005). Illuminating the Impact of Habitual Behaviors in Depression. *Chronobiology International*.

[27] Lin C., Chen I.-M., Chuang H.-H., Wang Z.-W., Lin H.-H., Lin Y.-H. (2023). Examining Human-Smartphone Interaction as a Proxy for Circadian Rhythm in Patients With Insomnia: Cross-Sectional Study. *Journal of Medical Internet Research*.

[28] Faul F., Erdfelder E., Lang A. G., Buchner A. (2007). G^*∗*^Power 3: A Flexible Statistical Power Analysis Program for the Social, Behavioral, and Biomedical Sciences. *Behavior Research Methods*.

[29] Kroenke K., Spitzer R. L., Williams J. B. W. (2001). The PHQ-9. *Journal of General Internal Medicine*.

[30] Liu S.-I., Yeh Z.-T., Huang H.-C. (2011). Validation of Patient Health Questionnaire for Depression Screening Among Primary Care Patients in Taiwan. *Comprehensive Psychiatry*.

[31] Health Promotion Administration (2018). Taiwan’s Obesity Prevention and Management Strategy. https://www.hpa.gov.tw/Pages/EBook.aspx%3Fnodeid%3D3813.

[32] Chuang H.-H., Lin Y.-H., Lee L.-A., Chang H.-C., She G.-J., Lin C. (2024). The Optimal Measurement Period of Actigraphy for Circadian Rhythm in Relation to Adiposity: A Retrospective Case-Control Study. *Sleep Medicine*.

[33] Chuang H.-H., Lin C., Lee L.-A., Chang H.-C., She G.-J., Lin Y.-H. (2024). Comparing Human-Smartphone Interactions and Actigraphy Measurements for Circadian Rhythm Stability and Adiposity: Algorithm Development and Validation Study. *Journal of Medical Internet Research*.

[34] Cole R. J., Kripke D. F., Gruen W., Mullaney D. J., Gillin J. C. (1992). Automatic Sleep/Wake Identification From Wrist Activity. *Sleep*.

[35] Gonçalves B. S. B., Adamowicz T., Louzada F. M., Moreno C. R., Araujo J. F. (2015). A Fresh Look at the Use of Nonparametric Analysis in Actimetry. *Sleep Medicine Reviews*.

[36] Jones S. E., van Hees V. T., Mazzotti D. R. (2019). Genetic Studies of Accelerometer-Based Sleep Measures Yield New Insights Into Human Sleep Behaviour. *Nature Communications*.

[37] Lin Y.-H., Wong B.-Y., Lin S.-H., Chiu Y.-C., Pan Y.-C., Lee Y.-H. (2019). Development of a Mobile Application (App) to Delineate, Digital Chronotype, and the Effects of Delayed Chronotype by Bedtime Smartphone Use. *Journal of Psychiatric Research*.

[38] Scrucca L., Fop M., Murphy T. B., Raftery A. E. (2016). mclust 5: Clustering, Classification and Density Estimation Using Gaussian Finite Mixture Models. *The R Journal*.

[39] Smagula S. F., Krafty R. T., Thayer J. F., Buysse D. J., Hall M. H. (2018). Rest-Activity Rhythm Profiles Associated With Manic-Hypomanic and Depressive Symptoms. *Journal of Psychiatric Research*.

[40] Smagula S. F., Krafty R. T., Taylor B. J., Martire L. M., Schulz R., Hall M. H. (2017). Rest-Activity Rhythm and Sleep Characteristics Associated With Depression Symptom Severity in Strained Dementia Caregivers. *Journal of Sleep Research*.

[41] Van Someren E. J. W., Riemersma-Van Der Lek R. F. (2007). Live to the Rhythm, Slave to the Rhythm. *Sleep Medicine Reviews*.

[42] Turek F. W. (2007). From Circadian Rhythms to Clock Genes in Depression. *International Clinical Psychopharmacology*.

[43] Germain A., Kupfer D. J. (2008). Circadian Rhythm Disturbances in Depression. *Human Psychopharmacology: Clinical and Experimental*.

[44] Baandrup L., Fasmer O. B., Glenthøj B. Y., Jennum P. J. (2016). Circadian Rest-Activity Rhythms During Benzodiazepine Tapering Covered by Melatonin Versus Placebo Add-on: Data Derived From a Randomized Clinical Trial. *BMC Psychiatry*.

[45] Monteleone P., Martiadis V., Maj M. (2011). Circadian Rhythms and Treatment Implications in Depression. *Progress in Neuro-Psychopharmacology and Biological Psychiatry*.

